# The Etiology, Incidence, Pathogenesis, Diagnostics, and Treatment of Canine Babesiosis Caused by *Babesia gibsoni* Infection

**DOI:** 10.3390/ani12060739

**Published:** 2022-03-16

**Authors:** Martina Karasová, Csilla Tóthová, Simona Grelová, Mária Fialkovičová

**Affiliations:** 1 Small Animal Clinic, University of Veterinary Medicine and Pharmacy, 04001 Košice, Slovakia; simona.grelova@student.uvlf.sk (S.G.); maria.fialkovicova@uvlf.sk (M.F.); 2 Clinic of Ruminants, University of Veterinary Medicine and Pharmacy, 04001 Košice, Slovakia; csilla.tothova@uvlf.sk

**Keywords:** dog, *Babesia gibsoni*, canine babesiosis

## Abstract

**Simple Summary:**

*Babesia gibsoni* is a parasite that causes the rupture of red blood cells in dogs. Although there is no natural, tick-borne transmission of this disease in Europe, it has become more common in European countries in recent years. Dogfighting breeds are predisposed to disease and they are a potential source of infection. Given the high popularity of these dog breeds in Europe and the participation of many dogs at sports competitions and shows, it is likely that the incidence of the disease in Europe may be higher than expected. The fact that the disease is mostly manifested as asymptomatic infection and that dogs of predisposed breeds are often imported from endemic areas, or they travel due to mating or competitions around the world, also contributes to this hypothesis.

**Abstract:**

*Babesia gibsoni* is one of the small *Babesia* species and the infection this pathogen causes is usually asymptomatic, which complicates the capture of potential parasite carriers. In endemic areas, especially in Asia, *B. gibsoni* occurs quite often due to direct transmission by way of a tick vector. Due to the absence of vectors, its occurrence is described only sporadically in Europe; but, it is increasingly occurring in predisposed, so-called fighting breeds, especially the American pit bull terrier. This review describes the etiology, incidence, clinical signs, pathogenesis, diagnostics, and treatment of *B. gibsoni* infection, with an emphasis on the clinical and laboratory peculiarities of the disease. As the treated dogs do not eliminate the parasite from the body—only reducing parasitemia and improving clinical signs—the treatment of *B. gibsoni* infection is a challenge in many cases, and its study therefore deserves great attention.

## 1. Introduction

Babesiosis is a parasitic infection caused by hemotropic protozoa of the genus *Babesia*, belonging to the family *Babesiidae*, order *Ixoplasmida*, class *Piroplasmea*, phyllum *Apicomplexa,* subkingdom *Alveolata,* and kingdom *Protozoa* [1]. The discovery of *Babesia* sp. was made in 1888 by the Romanian scientist Victor Babes, who identified *Babesia bovis* in cattle suffering from fever and hemoglobinuria [2]. Infections with *Babesia* sp. have complicated the lives of humans and domesticated cattle since ancient times. The second book of the Bible, Exodus 9:3, mentions that the cattle, horses, camels, donkeys, bulls, and sheep of the Egyptian Pharaoh Ramses II were gripped by a peculiar plague. The plague was manifested mainly by hemoglobinuria, and thus scientists believe that the disease would be babesiosis [3].

To date, more than 100 *Babesia* species have been scientifically described, which are specific to many species of mammals and birds [3]. In dogs, a large form of *Babesia* spp. is represented by *Babesia canis* (intra-erythrocytic merozoites measure 3–5 µm, which is at least half the diameter of the erythrocyte) and small forms of the disease are represented by *Babesia gibsoni, B. conradae,* and *B. vulpes* (merozoites measure 1–3 µm, which represents less than half the diameter of the erythrocyte). The dimensions of the *Babesia* parasite in relation to the size of the erythrocyte (approximately 7 µm) serve as a suitable aid for identifying the form of *Babesia* spp. in the affected dog [4]. 

*Babesia canis* includes three subspecies (*B. canis canis, B. canis rossi, and B. canis vogeli*), which have been identified based on their cross-immunity, serological testing, vector specificity, and molecular phylogeny. These subspecies are currently considered separate species [4,5].

Small *Babesia* spp. include *B. gibsoni* which comes from Asia, *B. conradae* [6], found in dogs in the western United States [7], and *B. microti*-like founded in Spain, renamed *Theileria annae,* and later *B. vulpes* [8,9,10]. *B. gibsoni* was originally divided into three subspecies: *B. gibsoni* (Asian genotype), *B. gibsoni* (California strain), and *B. gibsoni* (Spanish isolate) [11]. All three species were originally thought to be subspecies of *B. gibsoni*, but further molecular analysis and DNA sequencing showed that each could be marked as a separate species [8]. 

## 2. Occurrence

*B. gibsoni* is a tick-borne intracellular hemo-protozoan parasite of wild and domesticated canines [12]. In the available literature up to 2015, it is possible to find *B. gibsoni* divided into three subspecies. Therefore, for better orientation and to provide a comprehensive overview, all three currently separate species of small *Babesia* spp. are described in the etiology: *B. gibsoni* (Asian genotype), *B. conradae*, formerly *B. gibsoni* (California strain), and *B. vulpes*, syn. *Theileria annae*, formerly *B. gibsoni* (Spanish isolate).

For some time, *B. gibsoni* infection was observed only in certain parts of Asia, but it gradually spread throughout the Middle East and further to Africa, Europe, Brazil, North America, and Australia [9]. Although *B. gibsoni* (Asian genotype) is endemic in Sri Lanka (South Asia), Japan, Malaysia, Korea, and Egypt, it was widely diagnosed in the midwestern and the southeastern United States, and in Australia by the end of the 20th century [13,14,15,16,17]. The prevalence of the Asian genotype in Malaysia and Taiwan is approximately 17% [18], but it is more than 40% in Japan [19]. Many of the positive dogs from Japan, Taiwan, and the USA belonged to dogfighting breeds such as the American pit bull terrier, the American Staffordshire terrier, the Tosa Inu, and their hybrids [20]. It appears that although *B. gibsoni* (California strain), later renamed *B. conradae*, is endemic in California, the prevalence of this species has not increased among fighting breeds [21,22]. The Spanish isolate *B. gibsoni*, later renamed *B. vulpes*, is endemic in dogs in southwestern Spain [23]. According to recent studies, these three different subspecies are considered as individual species, thus, the following text deals only with *B. gibsoni* (Asian genotype) as an independent species of small *Babesia* spp. [8]. 

### Occurrence in Europe

**Figure 1:**
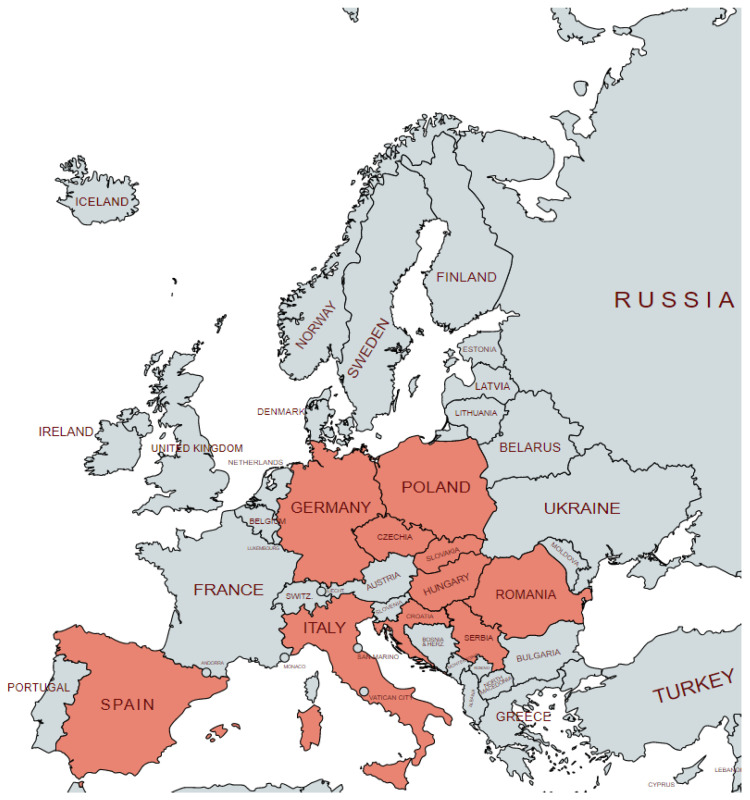
Occurrence of *Babesia gibsoni* in Europe.

Assessment of the *B. gibsoni* prevalence in Europe is particularly interesting due to the spread of the disease in areas where a natural vector does not occur and the assessment of a possible infection source often seems to be complicated. Figure 1 shows the documented prevalence of *B. gibsoni* infections in European countries. The first mention of *B. gibsoni* infections in Europe appeared in 1981 in Spain [24] and later in Italy [25], and the first clinical cases of infected dogs were documented in Spain [26] and in Hungary [27]. However, in all of these studies, *B. gibsoni* was diagnosed by only a blood smear examination, and it was identified by its size and its shape as a small *Babesia* sp. without further specification.

Molecular identification of *B. gibsoni* was first performed in Europe in 2003 by polymerase chain reaction (PCR) in a sick dog in Spain [28] and subsequently in Germany in two positive American pit bull terriers, as an autochthonous infection [29]. A further Spanish study of the vector-borne disease’s prevalence in 153 dogs from Barcelona confirmed the presence of *B. gibsoni* in 2% of the dogs examined [30].

In Croatia, a *B. gibsoni* prevalence survey was carried out on 848 randomly selected asymptomatic dogs, and it was found that six American pit bull terriers living in a village near the border with Bosnia and Herzegovina were positive (0.008% prevalence) [31]. 

In Italy, a case of babesiosis caused by *B. gibsoni* was described in an American pit bull terrier which was imported to the country from Croatia, and it was born to a bitch who was brought to Rome from the USA at the age of 4 months [32]. An Italian study conducted in 2018 confirmed a 0.2% *B. gibsoni* prevalence in 1311 randomly selected healthy dogs in southern Italy [33].

Romanian study confirms that dogfighting breeds are predisposed to the disease, as well as the American pit bull terrier being the most affected breed in the studies mentioned so far. PCR diagnostics was performed on 49 dogs with clinical signs compatible with canine babesiosis. There were 14 dogs positive for *B. gibsoni*: 13 dogs belonged to fighting breeds, 12 were American pit bull terriers, and one was an American Staffordshire terrier [34]. Another Romanian study looked at the prevalence of vector-borne diseases—including *B. gibsoni* infection—in 29 local pet dogs from Bucharest and in 187 stray dogs from Romania (*n* = 109) and Hungary (*n* = 78) that were imported to Germany for adoption. Although *B. gibsoni* was not found in local pet dogs, it was found in a group of stray dogs. Three dogs (0.03%) from Romania and one dog (0.01%) from Hungary were infected with *B. gibsoni* [35]. 

According to a study from Serbia, the prevalence of *B. gibsoni* was found in two dogs of fighting breeds (3.3%) among 60 dogs with clinical signs compatible with canine babesiosis. Both dogs were born in Serbia and they had never left the country. One of the dogs was a Tosa Inu with numerous scars on his head and neck, which were probably caused by dog bites. Even though the owner stated that the dog had a tick on it a few days prior, it is assumed that the bite wounds were related to the etiology of the disease in this case. The second dog was an 11-month-old American Staffordshire terrier, whose siblings and mother could not be traced, and thus potential vertical transmission was not confirmed [36]. Another Serbian study demonstrated the prevalence of *B. gibsoni* in 14 dogs (12.6%) out of 111 clinically healthy dogs that were kept primarily outdoors [37]. 

The first clinical case of *B. gibsoni* infection in Slovakia was published in 2016. The study describes the case of two American pit bull terriers from the same owner with severe clinical manifestations of parasitemia. Both dogs were born in Slovakia, but occasionally attended dog sport competitions in Hungary and the Czech Republic, where they encountered other dogfighting breeds which could have previously visited or come from an endemic country. Since the dogs come from the same household and sometimes they bite each other, transmission via biting should be considered [38].

In the Czech Republic, one case of an imported *B. gibsoni* infection in an American pit bull terrier, which came from Slovakia, has been confirmed so far. A positive dog was asymptomatic and identified through a study of the prevalence of babesiosis and hepatozoonosis in a randomized sample of 418 dogs (0.2% *B. gibsoni* prevalence) [39].

The first case of *B. gibsoni* infection identified in Poland was a clinical case of an infected American Staffordshire terrier [40], and another study detected its prevalence in 216 examined dogs, where three positive cases (1.4%) were found [41]. 

## 3. Transmission

So far, *B. gibsoni* infections have been reported in many non-endemic countries outside of Asia, especially in American pit bull terriers, Tosa Inu, and other dogfighting breeds. In these cases, there is convincing evidence that the dogs were used in dog fights and transmitted pathogens to each other by biting [42,43]. Fighting dogs are therefore considered a reservoir of this type of babesiosis and extensive injuries during fights, as well as the breeding of these dogs, support the spread of the disease without the presence of a vector. Due to the high popularity of these breeds and similar hybrids, it is assumed that *B. gibsoni* will eventually spread from countries where dog fights have been or are still being carried out (mostly illegally) to other countries even without the presence of a vector [44,45]. 

### 3.1. Vector Transmission

The main source of *B. gibsoni* transmission to dogs is ticks that carry prevalence infectious sporozoites in saliva. For the transmission of *B. gibsoni* to the dog host, the tick must be sucked into the skin for at least 48–72 h [46]. Most recovered dogs enter the premunition stage, which is an infectious immunity that maintains a balance between the dog’s immune response and the parasite’s ability to cause clinical disease. The host’s immune response protects it from a large multiplication of the parasite without being able to eliminate it. At this stage, infected dogs are at risk of recurrence and become a potential reservoir for tick-borne infection [13].

In the life cycle of *B. gibsoni*, two types of hosts are required, the tick and the dog. After swallowing the gametocytes by the tick, some of them transform into gametes, which fuse to form motile zygotes (gamogony phase). The zygote penetrates the peritrophic matrix (the semipermeable noncellular membrane surrounding the tick midgut lumen) and it invades the tick gut cells. Inside the epithelium, the zygote undergoes meiotic division, resulting in the production of kinetes. Kinetes migrate from the intestinal wall via the hemolymph system, and they penetrate to a variety of tissues and organs, including muscle cells, cells of Malpighian tubules, ovarian cells, and gonads. This process, which is repeated several times, continues in the ovaries as a sexual reproduction (transovarial transmission). Typically, only a small percentage of egg clusters laid by the tick will be infected with *B. gibsoni.* Intracellular kinetes change their form and they go through several division cycles (schizogony phase), forming secondary kinetes. These kinetes penetrate the cells of the salivary glands, where large multinucleated spore bodies form, eventually releasing thousands of small sporozoites (sporogony phase). 

Sporozoites are injected into a dog’s bloodstream along with tick saliva during blood-sucking (transstadial transmission), directly infecting erythrocytes and transforming into trophozoites. Asexual multiplication, i.e., binary fission of trophozoites, usually creates two, sometimes four, daughter cells that are called merozoites (merogony phase). Eventually, the host cell ruptures and the merozoites invade new erythrocytes again. This cycle continues for the entire life of the dog or until the dog’s immune system terminates the process. A few merozoites stop division and transform into gametocytes, the transient stage that infects the tick vector [47,48].

The most common vector of *B. gibsoni* in the endemic areas of India and Sri Lanka is the tick *Haemaphysalis bispinosa* in which both transovarial and transstadial transmission takes place, as well as *Rhipicephalus sanguineus* in India in which only transstadial transmission has been confirmed [49,50,51]. In Australia, *B. gibsoni* is transmitted by *Haemaphysalis longicornis* and widespread *R. sanguineus*; in Japan, infection is transmitted by *H. longicornis* [51]. The native vector of *B. gibsoni* in the USA is not known, neither *H. bispinosa* nor *H. longicornis* occur there. It is thought to be transmitted by *R. sanguineus* and *Dermacentor variabilis* ticks [6]. Although *H. longicornis* is an endemic tick in New Zealand, *B. gibsoni* infection does not occur in the country at all. This is due to the government’s strict quarantine measures for animal import and the need for a negative *B. gibsoni* test. Furthermore, *B. gibsoni* positive dogs are prohibited from entering the country [52]. 

### 3.2. Direct Transmission

Direct transmission of the disease can be accomplished through *B. gibsoni*-containing blood, for instance by blood transfusion; through the unhygienic use of contaminated instruments in mass ear cropping or the shortening of tails; and by repeated use of the same injection needle [16,53]. Direct transmission can also occur in dog fights and through bite wounds. This transmission is common among dogfighting breeds with a high incidence in American pit bull terriers, American Staffordshire terriers, Tosa dogs, and their hybrids, as these breeds have often been used for dogfighting [20,45]. This is evidenced in particular by studies on American pit bull terriers confiscated from dog fights and placed in shelters in the USA [16,42,43,45]. Aomori, a prefecture of the Japanese island Honshu, has a very rich dogfighting history, which is considered a part of the local tradition. Even today, dog fights are still popular there and they may even be legal [20]. In the United States, South Carolina has the highest number of illegal dog fights as presented by the media. 

Nowadays, there is still a lot of medialized evidence of illegal dog fights taking place in European countries. Owners of these dogs usually have dozens of dogs with a large pedigree history of fighting ancestors, so there is a high probability of breeding infected offspring and sharing disease to a healthy population of dogs through the sale of puppies locally and to foreign countries. Whereas dogfighting is illegal in many parts of the world, veterinarians should not rely on owners’ statements; scars on the skin, mainly on the head and front limbs, could quite likely be a result of dog bites. 

International sports shows and competitions of dogfighting breeds have a rich history in Europe. In addition to their exterior appearance, the dogs compete also in power, agility, and speed disciplines where various baits are used, especially toys and artificial skin. These baits are often shared among dogs in competitions, thus there exists a possibility of pathogen transmission by blood traces caused by chewing. 

### 3.3. Vertical Transmission 

Transplacental transmission was first confirmed in the experimental *B. gibsoni* infection of a bitch, 650 days before mating. At mating time, the bitch had a chronic infection with mild clinical signs and intraerythrocytic parasitemia in the peripheral blood below 0.001%. She was mated with an intact male. There was one stillborn puppy in the litter and four puppies that died on the 14th–39th day after birth. All of the puppies suffered from hypothermia, anemia, dysorexia, comatose, and subsequently died. An autopsy confirmed splenomegaly and hepatomegaly. In all puppies, DNA analysis confirmed the presence of *B. gibsoni*, which affirms transuterine disease transmission. The presence of *B. gibsoni* infection was also confirmed in samples taken from the brain, heart, lungs, and liver of a stillborn puppy. Immediately after birth, the puppies were exchanged with puppies of another intact bitch. In the puppies nursed by a bitch infected with *B. gibsoni*, PCR examination did not reveal the presence of parasitemia, which confirms that transmammary transmission by the milk of a positive mother is not possible in *B. gibsoni* infection. The transmission of *B. gibsoni* by the ejaculate of positive males has not been yet confirmed and it is not expected to occur [54].

## 4. Pathogenesis and Clinical Signs

The prepatent period—the time from infection to the demonstration of *B. gibsoni* piroplasms in the blood smears—is considered to be 7–11 days in dogs infected by *H. longicornis* and 2–40 days in the case of experimental infection. The incubation period—the time elapsed between exposure to a pathogenic organism and the appearance of the first symptoms—depends on the amount of the parasite load and it varies from 7 to 21 days.

Babesiosis caused by *B. gibsoni* is considered less pathogenic than babesiosis caused by *B. canis* and its course is mostly chronic, varying from a subclinical form to multiorgan failure and death [26,55]. The acute form of the disease is manifested by fever, lethargy, hemolytic anemia, and marked thrombocytopenia [56]. Dogs recovering from an acute infection become carriers of the pathogen, and the parasitemia persists for at least 38 months [57]. The chronic form is manifested by intermittent fever, lethargy, and weight loss and it can persist in the body for years [58].

Parasitic infection causes a systemic inflammatory response, which is considered to be a major aspect of the canine babesiosis pathophysiology and it contributes to a variety of clinical manifestations [59]. Cytokines, which are responsible for mediating and regulating all aspects of the immune response to infection, play an important role in inducing systemic inflammation [60]. The immunopathogenesis of canine babesiosis is not yet fully understood. The only cytokine identified, but associated with *B. canis* infection, is tumor necrosis factor alpha (TNF α), which was found in higher concentrations in dogs with higher peripheral parasitemia and more severe disease [61,62].

The clinical signs of *B. gibsoni* infection are variable; they do not depend only on the level of parasitemia but especially on the immune response of the infected dog [46]. The most severe disease occurs mainly in puppies and dogs younger than two years. Some dogs may have a mild fever, anorexia, depression, pale mucous membranes, lethargy, vomiting, loss of stamina, enlarged lymph nodes, and splenomegaly. Anemia and thrombocytopenia are most commonly detected by laboratory blood tests. In some cases, hemoglobinuria, hypoglycemia, acid-base imbalance [63,64,65], azotemia, proteinuria [66,67,68], elevated liver enzymes, and other organ dysfunctions are also associated. Complicated forms of babesiosis can manifest as coagulopathies, immune-mediated hemolytic anemia (IMHA), acute renal failure, hepatopathy, jaundice, pancreatitis, and multiorgan failure [69,70,71,72,73]. Skin changes associated with *B. gibsoni* infection are rare and they have been described in a few case studies. One of them manifested alopecia, dry skin exfoliation, hemorrhagic spots over the abdomen and the groin regions, interdigital ulcerative lesions, hyperkeratosis of the digital pads, brittleness of nails, and pododermatitis [74]. In another study, authors described cutaneous vasculitis with subsequently generalized alopecia, papules and erosions at the tips of the ears, skin ulcers, and necrosis of the limbs [75]. Another rare symptom is paraplegia which was described in the clinical case of *B. gibsoni* infection. In this case, a complete recovery from paraplegia was achieved by the end of the treatment [76].

The infection caused by *B. gibsoni* is often mild and mostly subclinical. However, *B. conradae* is more aggressive: it causes severe infection, and it can often be fatal [16,56]. In many cases, *B. vulpes* causes severe anemia and renal failure [9].

### 4.1. Hematological and Biochemical Alterations

Hematological alterations are usually manifested by regenerative hemolytic anemia together with poikilocytosis, polychromasia, anisocytosis, and thrombocytopenia [32,77]. Significant reductions in the red blood cell count and in the hemoglobin concentration are due to mechanical damage to erythrocytes during parasite migration out of the erythrocyte, intravascular hemolysis, and immune- or non-immune-mediated destruction of erythrocytes [6,78,79]. The erythrophagocytic activity of peripheral blood and bone marrow macrophages has been confirmed in *B. gibsoni* infected dogs, and it is considered another mechanism that exacerbates anemia [80]. The mechanism of thrombocytopenia has not been fully elucidated. One possible explanation is platelet sequestration in the spleen or immune-mediated platelet destruction and the development of disseminated intravascular coagulopathy (DIC) [79]. Leukogram alterations tend to be non-specific: sometimes marked or intermittent neutropenia [61,81] or sometimes neutrophilia and monocytosis, especially in acute disease [82]. Biochemical changes mainly include the elevation of hepatic and renal parameters and occasionally the elevation of pancreatic enzymes and hypoglycemia [81].

Electrophoretic alterations of serum proteins are characterized by a decrease and a complete disappearance of haptoglobin concentration within two weeks after infection; an increase in beta and gamma globulins; and a decrease in albumin in the acute disease [82,83]. Although the relative concentration of serum protein seems to be similar in various *Babesia* species, some differences exist among them. The mean values of the total proteins β1-, β2-, and γ-globulins are significantly higher in dogs infected with *B. gibsoni* than in dogs infected with *B. canis*. The relative concentrations of albumin, α1-, α2-globulins, and the A/G ratios are significantly lower in the *B. gibsoni* infected dogs [82]. 

The C-reactive protein (CRP) levels increase transiently on the third day after infection (19–20 mg/mL, normal up to 15 mg/mL); but, the increase is significant on day 13 and it peaks on day 15 (161 mg/mL), concomitant with the onset of peripheral parasitemia [59].

### 4.2. Ultrasonographic Alterations

Ultrasonographic alterations in the abdominal cavity usually complement the clinical pattern of the disease and the alteration of biochemical parameters. Increased cortical and medullary echogenicity, a relatively hypoechoic corticomedullary junction, and hypoechoic central medullary regions have been observed in dogs with anuria. Some dogs without clinical signs associated with the uropoetic system showed signs of a mild, transient increase in cortical and medullary echogenicity [81]. Diffuse enlargement of the spleen with mild parenchymal hypoechogenicity is a common finding, as the spleen is a major organ for immune defense against *B. gibsoni* infection [84].

### 4.3. Histopathological Alterations

Pathological changes mainly concern the spleen and the liver. According to some sources, hepatic degeneration occurs in all patients [85]. Affected dogs may show signs of enteritis, splenomegaly, renomegaly, and hepatomegaly. Congestion and edema are often observed in the lung tissue. Reactive lymphadenopathy, necrotizing arteritis, diffuse erythrophagocytosis, and extramedullary hematopoiesis occur in some cases. Microscopic changes observed in the kidneys include focal interstitial hemorrhages; glomerular atrophy to focal endothelial cell injury; necrotic and degenerative changes in tubular epithelial cells; diffuse nonsuppurative periportal and centrilobular hepatitis; interstitial fibrosis; focal desquamation of the tubular epithelium from the basement membrane; and membranoproliferative glomerulonephritis. Significant lymphocyte depletion occurs in the spleen at the same time as trabeculae proliferation and edema, hepatocyte lysis, and periportal fibrosis in the liver. The density of CD3+ lymphocytes in the hepatic sinuses increases significantly, and aggregates of large mononuclear cells with immunohistochemical properties of activated macrophages may be present in the central veins of the liver. Kupffer cells in the hepatic sinuses appear hypertrophic and prominent. The density of sinusoidal T cells, macrophages in the central veins, and the degree of Kupffer cell hypertrophy are more pronounced in dogs after splenectomy. The presence of multifocal IgM antibody deposits was immunohistochemically confirmed in the walls of the arteries changed by the inflammatory response and in the renal glomeruli. Intensive immunostimulation results in the activation and the expansion of the T and the B cell populations, macrophage activation, vasculitis, glomerulonephritis, and anemia, which are major pathological mechanisms in *B. gibsoni* infected dogs [86,87,88]. 

As in humans, severe clinical signs appear in dogs after undergoing splenectomy; therapeutic efforts are often ineffective, and the disease can be fatal for them. In these patients, the disease is severe and it is characterized by fever, anemia, jaundice, hemoglobinuria, and significantly decreased bilirubin and hematocrit levels below 11% [85,89].

## 5. Diagnostics

A correct diagnostic approach to *B. gibsoni* includes: an examination of the dog’s ancestry and travel history, analyzing epidemiological data in the area, determining whether the patient has undergone splenectomy or a blood transfusion, and determining whether the patient has been bitten by another dog. Based on this information, appropriate diagnostic methods should be performed [90].

In clinical veterinary practice, the most commonly used method to confirm the diagnosis is the microscopic detection of small *Babesia* sp. in blood smear, as this technique is easy to perform and faster than laboratory sample processing. The blood smear technique requires a certain amount of luck because it is not always possible to detect parasites in the blood smear, even in acute infection and in severe parasitemia. Therefore, laboratory analysis using PCR or immunological diagnostics is more convenient in *B. gibsoni* detection [91].

### 5.1. Microscopic Evaluation of a Blood Smear

Victor Babes was the first to identify *Babesia* sp. in a blood smear in 1888 when he searched for the cause of frequent bovine infections and discovered *B. bovis* [2]. The blood smear thus became the first method for the detection of *Babesia* spp. in clinical samples and it is still used in clinical practice as well as in laboratory diagnostics. 

For the detection of *B. gibsoni*, a blood sample is taken from the peripheral vein, in contrast to *B. canis*, where the sample is taken from the auricle capillary vein since *B. gibsoni* does not adhere to the vascular endothelium as does *B. canis* and *B. bovis*. The stained blood smear is observed under a microscope with the objective at 100× magnification. The finding of small intraerythrocytic merozoites, singular annular bodies measuring 1–3 μm is considered a positive finding of *B. gibsoni*. Merozoites are always smaller than the radius of the erythrocyte, which is a simple distinguishing feature from *B. canis* merozoites. Microscopic diagnostics is often complicated by the presence of different developmental stages of the parasite, such as trophozoites, which are characterized by a different, atypical shape and size, and this complicates and prolongs the diagnostic process.

The effectiveness of the blood smear technique depends on the microscopist’s experience and it requires adequate levels of parasites in the blood, which is often sufficient in acute disease but less so in chronic babesiosis—where a high probability of false-negative results exist. *Babesia* parasites could be easily confused with artifacts, and thus false-positive findings can occur [92].

### 5.2. Immunological Methods

Immunological techniques detect antibodies to *B. gibsoni* and they are considered effective and reliable, with high sensitivity but only moderate specificity due to cross-reactivity with *Babesia* spp. antigens and intact erythrocytes [12,93]. However, the disadvantage is that they rely on the presence of antibodies, which may develop over several days to weeks or may disappear completely after several months. Based on these facts, the effect of immunoassays in the case of acute infection or treated animals is quite limited and questionable. They are used especially in cases where the level of parasitemia is very low or not high enough to be detected by molecular methods [92].

If there is very low parasitemia to be detected by direct methods, it is appropriate to use the indirect fluorescent antibody test (IFAT) to detect antibodies and thus identify infectious asymptomatic carriers. This method was first used for *B. caballi* confirmation in chronically infected horses in 1964 [94]. Since then, this technique has been adapted to all *Babesia* species, including *B. gibsoni*, and it has quite a good specificity and sensitivity [12,95]. The IFAT is based on the recognition of parasitic antigens by serum antibodies. Bound antibodies are subsequently detected with a fluorochrome anti-Ig antibody (secondary antibody) [92]. The sensitivity and the specificity of IFAT in the detection of *B. gibsoni* antibodies are 93.1% and 88.9%, respectively. There is a small possibility to detect *B. gibsoni* in the early stage of infection. Thus, IFAT could be useful for diagnostics of chronically infected dogs with a significantly low level of parasitemia [91].

If it is necessary to evaluate a large number of samples at once, it is possible to use the enzyme-linked immunosorbent assay (ELISA), instead of IFAT, to save time. The advantages of the ELISA lie in its simplicity, non-subjectivity, high capacity for simultaneously evaluated samples, and higher specificity compared to IFAT. Recent ELISA tests use recombinant antigens and monoclonal antibodies to increase their specificity. Various *B. gibsoni* merozoite antigens have been evaluated for serodiagnosis of *B. gibsoni* infection in dogs. Recombinant BgSA1 is highly specific to *B. gibsoni* and it has proven efficacy in serodiagnostic assays in both acute and chronic stages of infection. ELISA tests for the detection of *B. gibsoni* using the recombinant BgSA1 merozoite antigen, namely double-antibody sandwich ELISA (DAS-ELISA), indirect ELISA, and dot-ELISA, were developed and their specificities were 81.6%, 84.2%, and 97.4%, respectively. These tests can reliably and with a high specificity identify the ongoing infection and monitor the parasitic load. No cross-reactivity with sera from dogs infected with *B. canis* was reported [96].

Immunochromatography is a rapid diagnostic test that can detect antibodies to specific antigens in a small amount of serum using specific antibodies and a recombinant antigen impregnated on nitrocellulose membrane-based test strips [97]. Immunochromatographic tests are quite convenient diagnostics because the method is rapid, easy, and simple. The cost of the test is lower than other techniques and it is performed within 15 min [96]. In recent years, several immunochromatographic tests have been developed with quite effective results, using recombinant P50, BgSA1, or thrombospondin-related adhesive protein [98,99,100].

### 5.3. Molecular Methods

Molecular methods for detecting *B. gibsoni* are aimed at detecting nucleic acids and they are quite useful even in cases where immunological methods cannot be used, for example, in the case of an acute or long-lasting chronic infection when antibodies may not be present in the body. A great advantage of using these methods is their high sensitivity and specificity [92]. The polymerase chain reaction has proven to be the best molecular method for *B. gibsoni* diagnostics so far, and it is considered the most accurate in the diagnosis of babesiosis caused by *B. gibsoni*.

The PCR technique was first used to diagnose *B. bovis*, *B. bigemina,* and *B. microti* in 1992 [92]. The PCR technique for the diagnosis of *B. gibsoni* was developed in 2001, and it is characterized by high sensitivity in the detection of specific parasitic DNA (0.000002% in a 2.5 µL blood sample). This technique can detect parasitemia much earlier than IFAT or microscopic examination of the blood smear, even in cases where the IFAT examination was negative [101]. Interestingly, in some very early or late stages of infection, it is not possible to detect parasites by PCR because *B. gibsoni* is thought to leave the circulatory system at some stages and undergo sequestration in other organs [102].

## 6. Treatment

To date, no treatment of *B. gibsoni* infection that would be able to completely eliminate parasites from the body has been described. In the case of small *Babesia* spp., frequent recurrences of the disease occur after treatment, even though the dog appears clinically healthy and the parasitemia falls below a level that can no longer be detected by PCR analysis. The effect of drugs are only in mortality reduction and the alleviation of the clinical signs of the disease [13]. The owner of the dog treated for *B. gibsoni* infection must be prepared for the risk of recurrence of the disease and they must reckon with the fact that the dog becomes a source of infection and a potential reservoir [3].

### 6.1. Imidocarb Dipropionate

Imidocarb dipropionate is an aromatic diamidine and its effect is explained by several mechanisms. One possibility is that it blocks the uptake of inositol into erythrocytes infected with *Babesia* sp. and subsequently causes their starvation [103]. The second explanation is that it combines with *Babesia* DNA, causing nucleic acid damage while blocking cell repair and further replication [104]. Imidocarb dipropionate is excreted by the kidneys and the liver.

Imidocarb dipropionate is commonly used in Europe to treat parasitosis caused by *B. canis*; but, at the same time, its effect has been demonstrated in small *Babesia* spp., including *B. gibsoni*. However, the effect of imidocarb dipropionate in *B. gibsoni* treatment is unsatisfactory and its use is therefore not recommended [6,105,106].

The therapeutic dose of imidocarb dipropionate is 6.6 mg/kg of body weight, intramuscularly or subcutaneously in 2 doses at 14-day intervals. The side effects of imidocarb dipropionate in dogs include painful injection and parasympathomimetic symptoms such as salivation, vomiting, and nasal discharge. These effects can be alleviated by the administration of atropine premedication at a dose of 0.05 mg/kg of body weight. Other side effects that occur less frequently are dyspnea, general weakness, fever, muscle cramps, diarrhea, renal tubular or hepatic necrosis, anaphylactic reaction, and an inflammatory reaction to ulceration at the injection site [107].

### 6.2. Diminazene Aceturate

Diminazene aceturate is an aromatic diamidine, similar to imidocarb dipropionate. The mechanism of action on *Babesia* spp. is not fully elucidated, but it is thought to disrupt parasite DNA synthesis and aerobic glycolysis [104]. Diminazene aceturate is excreted via the kidneys and the liver. 

Diminazene aceturate is currently not an approved drug in many countries, including the United States, due to the occurrence of severe side effects. In South Africa, however, it remains the drug of the first choice in the treatment of canine babesiosis caused by *B. rossi* [108]. The dose of the active substance is 3–5 mg/kg of body weight, in two applications at 7-day intervals [104,108,109]. The therapeutic effect of diminazene aceturate on *B. gibsoni* infection is good, but it is more effective in large *Babesia* species. On the other hand, it could be toxic, causing serious neurological damage even in therapeutic doses. In the case of repeated low doses over a short period, its accumulation in the body can cause severe toxicosis affecting the kidneys, brain, and liver. The side effects that often occur in animals after the administration of a therapeutic dose include indigestion (vomiting, diarrhea), pain and inflammation at the injection site, transient hypotension, and sometimes severe neurological symptoms including weakness, ataxia, convulsions, paralysis, and death, especially in dogs [78,104,110]. Another disadvantage of using this drug in the treatment of babesiosis is the high percentage of disease recurrences [93,110].

In the past, diminazene aceturate was withdrawn from the market in Japan, but today it is still used there for *B. gibsoni* treatment. To reduce the risk of side effects and recurrence, diminazene aceturate is used at lower doses (2 mg/kg of body weight in three subcutaneous applications every 48 h), in combination with clindamycin (25 mg/kg of body weight, orally every 12 h for 21 days) [111]. Its effect on *B. gibsoni* infection in patients who have undergone splenectomy is insufficient and its use in these cases is not recommended [112]. 

### 6.3. Atovaquone

Atovaquone is a synthetic hydroxy-1,4-naphthoquinone and its effect is antiprotozoal. Hydroxynaphthoquinones selectively block protozoal mitochondrial electron transport, causing the inhibition of pyrimidine and adenosine triphosphate synthesis [113]. 

Atovaquone is used in the treatment of *B. gibsoni*, *B. conradae*, and *B. vulpes* at a dose of 13.3 (or 13.5) mg/kg of body weight, orally with a fatty meal (maximizing drug absorption) every 8 h, in combination with azithromycin at a dose of 10 mg/kg of body weight, orally every 24 h; both drugs are administered together for 10 days [105,106,114,115,116]. Atovaquone is available in Malarone^®^ in combination with proguanil hydrochloride and it is commonly used in the prevention and treatment of malaria in humans. Proguanil hydrochloride causes side effects in dogs in the form of digestive problems, and therefore it is more appropriate to use atovaquone as a separate drug (Mepron^®^, Atovaquone^®^). However, such a product is not registered in many European countries at this time and one of its disadvantages is its high price. 

The therapeutic effect of atovaquone has also been demonstrated in the treatment of *B. divergens* in cattle, *B. microti* in humans [117,118,119], a wide range of protozoan pathogens in humans such as *Plasmodium* spp. and *Toxoplasma gondii* [114], as well as in the treatment of *B. gibsoni* infection in dogs [120]. In combination with azithromycin, it has been shown to reduce *B. microti* parasitemia in humans as well as in hamsters and *Cytauxzoon felis* parasitemia in cats [121,122]. Many authors agree that the effect of atovaquone alone is not sufficient due to frequent recurrences of the disease and the low sensitivity of protozoan parasites to treatment [119,123,124]. By applying atovaquone as monotherapy, mutations in the cytochrome b (CYTb) gene and subsequent amino acid substitution of atovaquone binding sites can often occur, causing atovaquone resistance [125].

The simultaneous use of atovaquone with azithromycin has an additive or synergistic therapeutic effect, while the administration of atovaquone alone causes a recurrence of clinical signs. In the treatment of *B. gibsoni* infection in dogs, the combination of atovaquone (+/− proguanil hydrochloride) and azithromycin is considered to be the most effective, reducing parasitemia below the level that can be detected by PCR—although not in all cases. However, there are frequent recurrences of the disease, despite the good efficacy of this treatment [114,126,127,128,129].

### 6.4. Antibiotics

Many antibiotics have been tried in the treatment of *B. gibsoni* infection, but their therapeutic effects have not been satisfactory in any case. Some studies describe the administration of antibiotics as monotherapy, such as doxycycline [130], clindamycin (11 mg/kg of body weight intravenously every 24 h for 10 days or 25 mg/kg of body weight orally every 12 h for 14 days) [79,131], or enrofloxacin (in vitro experiment) [132]. Their therapeutic use as monotherapy had the effect of reducing clinical signs and partially adjusting the blood parameters, but in most cases, there was no absolute cure as the parasitemia disappeared or there was a frequent recurrence of the disease within a short period after the end of treatment. In many studies, only blood smears—not PCR tests—were used to monitor the treatment response; thus, the success rate of the treatment is likely to be much lower than reported as *B. gibsoni* is often not detectable in blood smears, even in cases of high parasitemia. 

Azithromycin is a macrolide antibiotic that can bind to the 50S ribosome subunit of prokaryotes and thus inhibit mediator ribonucleic acid (mRNA) translation and bacterial protein synthesis. At the same time, azithromycin also has an antiprotozoal effect due to its specific activity on apicoplast parasites, including *Babesia* spp. Apicoplasts are four-membrane cell organelles of *Apicomplexa* parasites that have lost the ability to carry out photosynthesis; thus, parasites are unable to survive without them because metabolic processes take place in them [133,134]. The effect of azithromycin has been demonstrated in the treatment of *Plasmodium falciparum* [135] as well as *Toxoplasma gondii* [136].

Azithromycin’s side effects include an irritated stomach, stomach cramps, nausea, diarrhea, and rare instances of vomiting [137]. 

### 6.5. Drug Combinations

In addition to the combinations mentioned, other variants of therapeutic protocols have been used in the treatment of *B. gibsoni* infection. One study describes the effects of a triple-drug combination: clindamycin (30 mg/kg of body weight orally every 12 h), diminazene aceturate (3.5 mg/kg of body weight intramuscularly once), and imidocarb dipropionate (6 mg/kg of body weight subcutaneously once, 24 h after diminazene application). In 13 monitored dogs, there was no complete clinical cure in 11 of the dogs and relapses occurred shortly after the end of the treatment [138].

The effect of the antibiotic combination of doxycycline (7–10 mg/kg of body weight orally every 12 h), enrofloxacin (2–2.5 mg/kg of body weight orally every 12 h), and metronidazole (5–15 mg/kg of body weight orally every 12 h) was successful in 83.3% of cases after 6 weeks of treatment of the *B. gibsoni* infection. However, with the addition of diminazene aceturate (3 mg/kg of body weight intramuscularly every 7 days), the effect increased to 85.7%. The treatment was given to patients for several weeks, depending on their responses and blood test results [139]. 

In another study, Suzuki et al. studied the effect of a combination of clindamycin (25 mg/kg of body weight orally every 12 h), metronidazole (15 mg/kg of body weight orally every 12 h per day), and doxycycline (5 mg/kg of body weight orally every 12 h), where three of the four dogs examined showed the elimination of parasitemia and the fourth dog had a recurrence of the disease in a short time [109].

### 6.6. Supportive Care

Supportive care should be based on a thorough assessment of the patient’s clinical condition and it should be used in cases of moderate to severe infection to alleviate the clinical signs of the disease and to reduce the patient’s mortality risk. The choice of supportive care depends on an assessment of the clinical signs and the blood test results. For example, in respiratory collapse, oxygen therapy, and antiemetics should be used to prevent vomiting and the subsequent aspiration of gastric content into the lungs. If the patient is stable and they do not require hospitalization, the treatment plan should focus only on antiprotozoal therapy. 

Intravenous fluid therapy could be used in the cases of shock, severe infection, dehydration, intravascular hemolysis, hemoglobinuria, and impaired kidney function. The most suitable intravenous solutions are crystalloids, together with solutions to correct acid-base imbalance and electrolyte abnormalities [140].

Blood transfusion is indicated in patients with severe anemia (hematocrit <15%) and severe dyspnoea or tachypnoea. The degree of parasitemia is not an essential criterion when considering transfusion, as it often does not correlate with the patient’s degree of anemia. 

The use of immunosuppressive therapy in dogs with IMHA or thrombocytopenia is considered controversial, as this condition is always related to the infectious nature of the disease. However, if the patient does not respond to antiprotozoal treatment, it is recommended to administer prednisone at a dose of 2 mg/kg of body weight every 24 h in patients with moderate to severe disease. Corticosteroid therapy is contraindicated in patients who have undergone a splenectomy [141].

## 7. Prevention

In endemic areas where the transmission of *B. gibsoni* by ticks occurs, the use of repellent antiparasitic preparations to prevent ticks from attaching to the dog is necessary and the best form of prevention. Once attachment occurs, the tick should be removed as soon as possible, since the potential infection could manifest approximately 48 h after the tick bite. According to a retrospective study of dogs traveling to endemic areas, pet owners underestimate antiparasitic prophylaxis of the dog and thus many of them return home with "exotic" diseases, including *B. gibsoni* infection (10% incidence) [142].

Merozoites circulating in the blood of an infected dog could be a source of infection for a patient who receives a blood transfusion from a *B. gibsoni* infected donor. As the disease is often asymptomatic, the blood of the donor should be examined for anemia before a blood transfusion, ideally by PCR or at least blood smear examination to rule out the presence of parasitemia. This is particularly important in endemic areas, where *B. gibsoni* is common. In non-endemic areas, it is necessary to pay attention to the travel history of the donor and proceed with increased caution for American pit bull terriers and American Staffordshire terriers as well as their hybrids, especially in the case of mild anemia. In the case of a recipient who has undergone a splenectomy in the past, the donor should be selected carefully as *B. gibsoni* infection can be fatal in these patients [53,143].

Since *B. gibsoni* infection is also vertically transmissible, bitches with the previous infection should be excluded from breeding. A bitch that has been treated for *B. gibsoni* infection and has a negative PCR test at the time of mating is considered to be an asymptomatic carrier and the disease will be transmitted to a part of the litter. Pregnancy itself can also cause immunosuppression and the recurrence of infection in a bitch [54].

One of the possible transmission paths is through dog biting, which needs to be prevented, especially in predisposed breeds [102].

### 7.1. Vaccination

Developed from the *B. canis* and the *B. rossi* parasitic antigens, the Pirodog® (Merial) vaccine is currently available on the European market, with the effect of shortening the disease duration and reducing the severity of clinical signs. Although the vaccine does not prevent infection, it appears to block the development of the pathological process to some extent. The level of preventive protection is highly variable (70–100%) and it has no effect against other *Babesia* spp. [144,145,146]. Vaccines against *B. gibsoni* are the subject of studies and their development is based mainly on the use of recombinant antigens and DNA [147,148,149].

### 7.2. Zoonotic Potential

To date, the zoonotic potential of any *Babesia* sp. that primarily affects dogs or cats has not been demonstrated sufficiently [90]. Nevertheless, several cases of human infection with *B. gibsoni* have been documented. Two cases have been reported in people who underwent a splenectomy [150,151]. Another case of *B. gibsoni* infection has been reported in California [21]. In these cases, the etiology of the disease and the source of the infection or the vector are not clear. 

## 8. Conclusions

This review described in detail the etiology and pathogenesis of the disease, with an emphasis on pointing out the possible causes of *B. gibsoni* in Europe, where the disease vector does not naturally occur. In recent years, the prevalence of the disease has also increased on the European continent due to travel with dogs, the import of interesting dog breeds from exotic countries, and illegal dog fights. Since the disease is usually asymptomatic, infected dogs become hidden carriers of the disease with the risk of transmission, especially during blood transfusions. Therefore, it is important to know the etiology and symptomatology of the disease, the diagnostic options, and the proper approach for treating the disease. The treatment options currently in use do not provide an adequate therapeutic response and infected dogs have frequent disease recurrences, so it is necessary to continue in the study of treatment for *B. gibsoni* infections and search for new therapeutic protocols.
